# CircCAMSAP1 promotes hepatocellular carcinoma progression through miR‐1294/GRAMD1A pathway

**DOI:** 10.1111/jcmm.16254

**Published:** 2021-01-23

**Authors:** Zongjiang Luo, Libai Lu, Qianli Tang, Wang Wei, Pengyu Chen, Yichen Chen, Jian Pu, Jianchu Wang

**Affiliations:** ^1^ Department of Hepatobiliary Surgery Affiliated Hospital of Youjiang Medical University for Nationalities Baise China; ^2^ Clinic Medicine Research Center of Hepatobiliary Diseases Affiliated Hospital of Youjiang Medical University for Nationalities Baise China

**Keywords:** CircCAMSAP1, circRNA, GRAMD1A, hepatocellular carcinoma, miR‐1294

## Abstract

Hepatocellular carcinoma (HCC) is one of the most common cancers with high prevalence and mortality, and it has brought huge economic and health burden for the world. It is urgent to found novel targets for HCC diagnosis and clinical intervention. Circular RNA (circRNA) has been reported to participate in many cancer progressions including HCC, suggesting that circRNA might paly essential role in HCC initiation and progression. Our study aims to found that potential circRNA participates in HCC development and its underlying molecular mechanisms. We obtained three pairs of HCC tissues and its adjacent normal tissues data from GEO DataSets. MTT, cell colony, EdU, wound‐healing, transwell invasion and mouse xenograft model assays were used to demonstrate the biological functions of *circCAMSAP1* in HCC progression. Furthermore, we conducted bioinformatics analysis, AGO2‐RIP, RNA pull‐down and luciferase reporter assays to assess the association of *circCAMSAP1‐miR‐1294‐GRAMD1A* axis in HCC cells. The expression of *circCAMSAP1* was up‐regulated in HCC tissues compared with its adjacent normal tissues. Up‐regulation of *circCAMSAP1* promoted HCC biological functions both in vitro and in vivo. The promotive effects of *circCAMSAP1* on HCC progression function through *miR‐1294/GRAMD1A* pathway. *CircCAMSAP1* was up‐regulated in HCC tissues, and *circCAMSAP1* up‐regulated *GRAMD1A* expression to promote HCC proliferation, migration and invasion through *miR‐1294*. *CircCAMSAP1* might be a potential prognosis and therapeutic target for HCC.

## 1 INTRODUCTION

Hepatocellular carcinoma (HCC), as one most common type of primary liver cancer, ranks the third cause of leading cancer‐related deaths worldwide.^1^ The poor diagnostic strategy, frequent metastasis, lacking efficient treatment and high rate of relapse leads to the high mortality of HCC and threatens the world healthcare system.^2^ Despite the risk factors including hepatitis C virus infection, obesity, alcohol and metabolic disorders,^3^, ^4^ it is imperative to elucidate the underlying genetic and epigenetic mutations of hepatocarcinogenesis and found out the novel molecular target for diagnosis and clinical intervention. Recently, an increasing number of studies demonstrated that circular RNA (circRNA) is involved in the initiation and progression of HCC tumorigenesis,^5^, ^6^, ^7^ suggesting that circRNA may be an important role in HCC prognosis and intervention.

CircRNA is characterized by its close loop structure, which is more stable than other RNAs and is abundantly expressed in human tissues or serum.^8^, ^9^ In the past two decades, the biological functions of circRNA have been in‐depth studied in cancer including HCC.^10^, ^11^ Wang et al demonstrated the promotive function of *circRHOT1* in HCC progression,^12^ Zhang et al revealed that *circTRIM33‐12* suppresses HCC progression via sponging *microRNA‐191*,^13^ and Yu et al elucidated the biological functions of circular RNA *cSMARCA5* in HCC development.^14^ Those researches suggest that the biological function of circRNA in HCC development demands further study.

The molecular functions of circRNA have been elucidated, such as the gene translation, protein binding. Remarkably, it has been widely reported that circRNA acts as a sponge for microRNA (miRNA) to regulate biological progress.^15^, ^16^, ^17^ By acting as a competitive endogenous RNA for miRNA, circRNA restricts the biological function of miRNA, such as binding and regulating mRNA, and regulates epigenetic progress.^18^ Moreover, the functions of circRNA‐miRNA axis in HCC progression have widely reported.^19^, ^20^ The above evidence indicates that the underlying molecular mechanisms of circRNA in HCC initiation and progression may mainly act as miRNA sponge, but this proposal needs more study to further confirm.

In this study, we aim to found the potential circRNA participates in HCC initiation and progression. By performing the GEO Datasets analysis, we found that the expression of circular RNA *circCAMSAP1* was up‐regulated in three pairs of HCC tissues. Next, we performed a serial in vitro experiment and we found the promotive effect of *circCAMSAP1* in HCC biological progression. Furthermore, we performed bioinformatics analysis, AGO2‐RIP, RNA pull‐down and dual‐luciferase reporter assays, and we found that *circCAMSAP1* promotes HCC development via *miR‐1294/ GRAMD1A*. Our study may provide new insight into cricRNA research in HCC development and a potential therapeutic target for HCC intervention.

## 2 MATERIALS AND METHODS

### 2.1 Cell culture and transfection

HCC cell lines HepG2, Hep3B, Huh‐7, SMMC‐7721, MHCC‐97H and normal liver cell Lo‐2 were purchased from the Cell Resource Center of Shanghai Institutes for Biological Sciences. Cells were cultured in DMEM (Gibco) with 10% FBS (Gibco) at 37°C in a humidified atmosphere with 5% CO^2^. All plasmids and shRNAs were commercially obtained from GeneChem. Lipofectamine 2000 (Invitrogen) was applied for all transfection according to the manufacturer's instructions. *CircCAMSAP1* overexpression plasmids were constructed using human *circCAMSAP1* cDNA, which was synthesized by GenePharma, and subjected into the pGLV5‐ciR vector (GenePharma). The sequences are shown as follows: ShcircCAMSAP1#1: TCAACAAGATAACATCCCT, ShcircCAMSAP1#2: GGATCAACAAGATAACATC, ShcircCAMSAP1#3: ACAAGATAACATCCCTGAG and circCAMSAP1 RNA pull‐down probe: ACTTCCTATCTCACTCCTCCCTAATGACCTAATTCAGTCTCATGGCTAGAAATACC.

### 2.2 Quantitative real‐time PCR

TRIzol (Invitrogen) was used for total RNA isolation. Total DNAs were synthesized by conducting the PrimeScript RT reagent Kit (Takara Bio). Real‐time PCR was performed by using Real Master Mix (SYBR Green). RNase R (Epicentre) was used to digest RNAs, remove the liner RNAs and enrich the circular configuration. The primers used in this study are as follows: *circCAMSAP1* forward: GTGTCAAGCGCTTCTCAACG, reverse: GCTGGACAGGAGAAGCTTGA; *miR‐1294* forward: TGTGAGGTTGGCATTGTTGTCTGT, reverse: GTGCAGGGTCCGAGGTATTC; *GRAMD1A* forward: ACACAATGGGCTACTGTGAGG, reverse: GGCTTGGTCTCGATGCTACT; and *GAPDH* forward: CGCTCTCTGCTCCTCCTGTTC, reverse: ATCCGTTGACTCCGACCTTCAC. 2^−ΔΔCt^ method was used to calculate the relative expression levels of *miR‐1294* and *GRAMD1*. Experiment was repeated three times.

### 2.3 Colony formation assay

Twenty‐ four hours after transfection, cells were collected and seeded in a 6‐well plate (1 × 10^3^ cells for each plate) in DMEM with 10% FBS for 10 days. Then, PBS was used to wash cells for three times and cells were incubated with methanol (Beyotime) and stained with 0.1% crystal violet (Invitrogen) half an hour. Cells number >50 were counted. Experiment was repeated three times.

### 2.4 MTT assay

Cell proliferation ability was detected by using MTT kit (Trevigen) following the manufacturer's protocol. Briefly, cells were subjected into a 96‐well plate for 24 hours (1 × 10^3^ cells for each plate). Then, a flow cytometer was used to analyse cells, and CellQuest software (BD) was applied to analyse results. Experiment was repeated three times.

### 2.5 5‐Ethynyl‐2′‐deoxyuridine (EdU) assay

Cell‐Light EdU DNA Cell proliferation kit (RiboBio) was used to detect cell proliferation ability according to the manufacturer's instructions. Forty‐eight hours after transfection, cells (1 × 10^4^ cells for each group) were treated with 50 mmol/L EdU 2 hours. DAPI was used to stain nucleic acids, and cells were stained with Apollo Dye Solution. ImageJ (NIH) was used to calculate the cell proliferation rate. Experiment was repeated three times.

### 2.6 Transwell invasion assay

The upper chamber was fixed with 100 µL serum‐free suspension, and the bottom chamber was fixed with 600 µL DMEM with 10% FBS. Twenty‐ four hours after incubation, cells (1 × 10^3^ cells for each group) were treated with crystal violet staining. Penetrating cells were calculated with 5 randomly picked fields, and calculation repeated three times. Experiment was repeated three times.

### 2.7 FISH assay

FISH kit (RiboBio) was used to detect the location of *CircCAMSAP1* in HCC cell following the manufacturer's protocol. Briefly, cells (1 × 10^3^ cells for each group) were subjected to pre‐hybridization solution for 30 min. Then, at a 20 µmol/L concentration of hybridization solution, probes were dissolved and subjected to slides and hybridized 12 hours. Next, slides were washed by using saline sodium citrate and subjected to DAPI for 15 min. A confocal microscopy was applied to analyse results. Sequences of *circCAMSAP1* probe: CAGGGATGTTATCTTGTTGATCCAGAAC. Experiment was repeated three times.

### 2.8 Luciferase reporter assay

The *miR‐1294* sequences containing wild‐type or mutant binding sites of circCAMSAP1/GRAMD1A were cotransfected into pmirGLO vectors (Promega). With pRL‐TK vector (Promega), the luciferase vectors and miR‐1294 mimics were cotransfected into SMMC‐7721 cells (1 × 10^4^ cells for each group). Luciferase activity results were measured by using the Dual‐Luciferase Reporter Assay System (Promega). Experiment was repeated three times.

### 2.9 RNA immunoprecipitation assay

The Magna RIP RNA‐binding protein immunoprecipitation kit (Millipore) was applied to perform RIP assay following the manufacturer's protocol, and anti‐*AGO2* (#03‐110, Millipore) was used. qRT‐PCR assay was performed to analyse the purified RNAs. IgG was used as an isotype control. Experiment was repeated three times.

### 2.10 RNA Pull‐down assay

Biotin‐labelled *circCAMSAP1* and control probes were synthesized by GeneChem. Collectively, cells were lysed using co‐immunoprecipitation buffer (Beyotime), and the cell lysis was subjected to high amplitude for 30 cycles. *CircCAMSAP1* probe‐streptavidin beads (Life) were subjected to cell lysis overnight. And, TRIzol reagent was applied for RNA isolation. qRT‐PCR was performed to analyse purified RNAs. Experiment was repeated three times.

### 2.11 In vivo mouse xenograft

The animal experiment was approved by the Committee for Animal Care and Use of Affiliated Hospital of Youjiang Medical University for Nationalities. NOD/SCID mice (8 weeks old) were randomly divided into two groups (n = 6). NOD/SCID mice were inoculated subcutaneously with SMMC‐7721 cells (1 × 10^7^ per tumour) pre‐transfected with sh‐NC or sh‐circCAMSAP1. Tumour volumes were measured every 5 days.

### 2.12 Statistical analysis

SPSS 19.0 (IBM) was conducted to perform statistical analysis. Student's *t* test or one‐way ANOVA was applied to test the differences among groups. Data were presented as mean ± SD *P* < .05 was considered as statistical significance.

## 3 RESULTS

### 3.1 CircCAMSAP1 is highly expressed in HCC tissues

**FIGURE 1:**
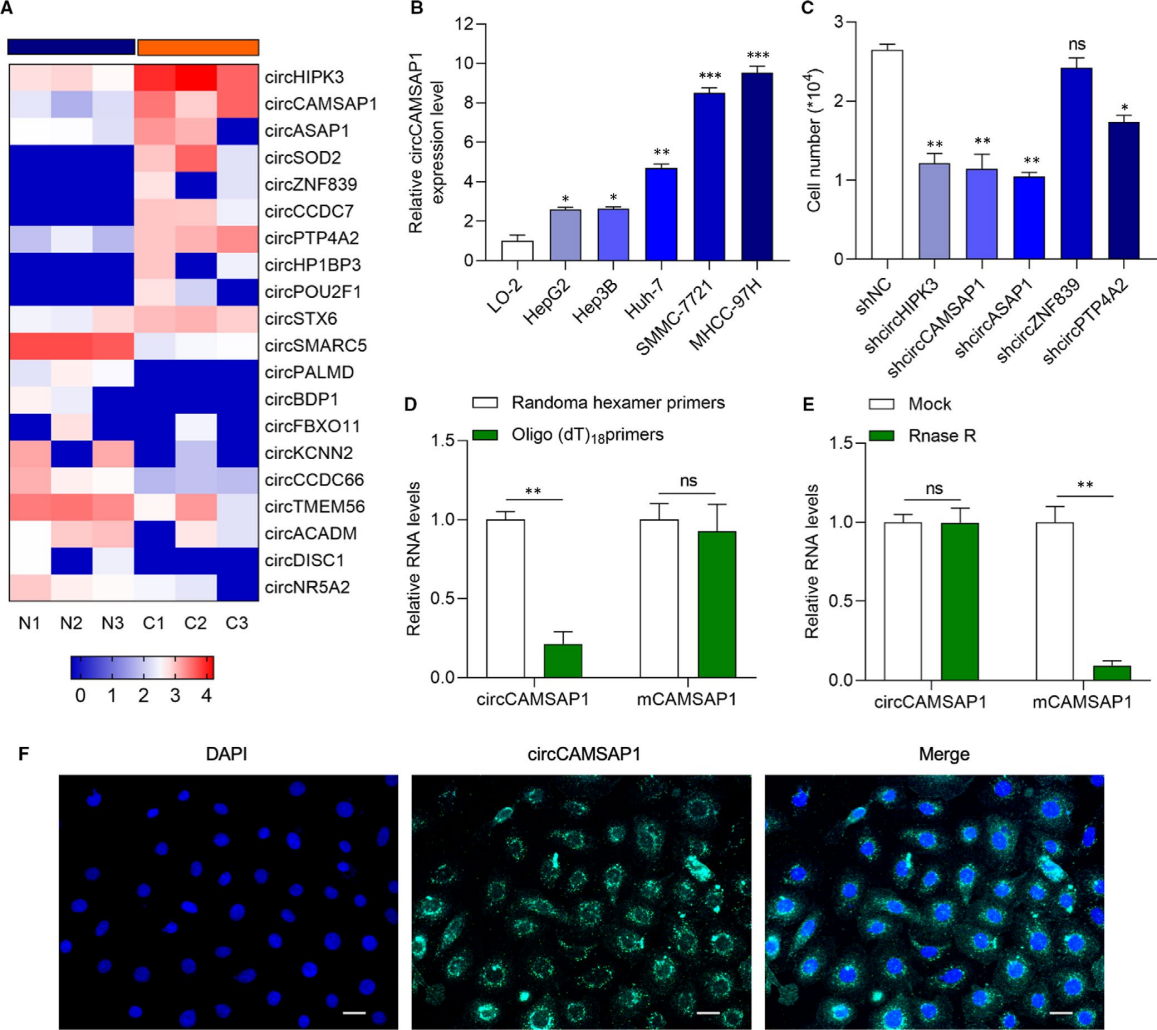
CircCAMSAP1 is highly expressed in HCC tissues. A, Heatmap of the differentially expressed circRNAs in three pairs of HCC tumour tissues and normal tissues (NT) from GEO DataSets (GSE125469), which is from the initially published data. Unit of the data displayed in the heatmap is analysed using Logged CPM. The download source of GSE125469 is https://www.ncbi.nlm.nih.gov/geo/query/acc.cgi?acc=GSE125469. B, qRT‐PCR analysis of circCAMSAP1 expression was increased in HCC cell lines (HepG2, Hep3B, Huh‐7, SMMC‐7721 and MHCC‐97H) compared with normal hepatocellular epithelial cells (LO‐2). C, SMMC‐7721 cell numbers in indicated shRNA‐treated group compared to negative control (NC) control group after shRNA treatment by applying cell colony assay. D, The relative RNA levels in SMMC‐7721 were analysed by RT‐qPCR and normalized to the value using random hexamer primers. E, The relative RNA levels in SMMC‐7721 were analysed by RT‐qPCR and normalized to the value detected in the mock group. F, RNA FISH for circCAMSAP1 in SMMC‐7721. Nuclei were stained with DAPI. Scale bars, 100 µm. All experiments were repeated at least three times. *P < .05. **P < .01. ***P < .001

To identify the potential circRNA which participates in the progression of HCC, RNA sequencing analysis of three pairs of HCC tissues and normal tissues (NT) samples data from GEO DataSets (GSE125469) were used.^21^ Heatmap of the differentially expressed circRNAs is presented in Figure 1A. The role of *circHIPK3* and *circASAP1* has been investigated in HCC.^23^, ^24^ We selected *circCAMSAP1*, which is found to participate in colorectal cancer progression,^26^, ^27^ for further study. It showed that *circCAMSAP1* was significantly overexpressed in HCC tissues in comparison with normal tissues. Then, we chose HCC cell lines SMMC‐7721 and MHCC‐97H, which abundantly expressed *circCAMSAP1* for further study (Figure 1B). Next, we tested the proliferation ability of HCC cell line SMMC‐7721 transfected with sh‐circRNA and its negative control, respectively. It was found that the down‐regulation of circCAMSAP1 significantly inhibited SMMC‐7721 proliferation (Figure 1C). Those results indicate that circCAMSAP1 may involve in the initiation and progression of HCC. Random hexamer or oligo (dT)18 primers, which could amplify mRNA but not circRNA, were used in the reverse transcription experiments. As shown in Figure 1D, the expression of liner RNA *mCAMSAP1*, but not circular RNA *circCAMSAP1*, was obviously up‐regulated. The RNase R treatment also showed that *circCAMSAP1* was more stable than *mCAMSAP1* (Figure 1E). RNA FISH assay showed that *circCAMSAP1* mainly located in the cytoplasm (Figure 1F). *CircCAMSAP1* might participate in HCC progression.

### 3.2 Knockdown of circCAMSAP1 inhibits HCC cell proliferation, migration and invasion

**FIGURE 2:**
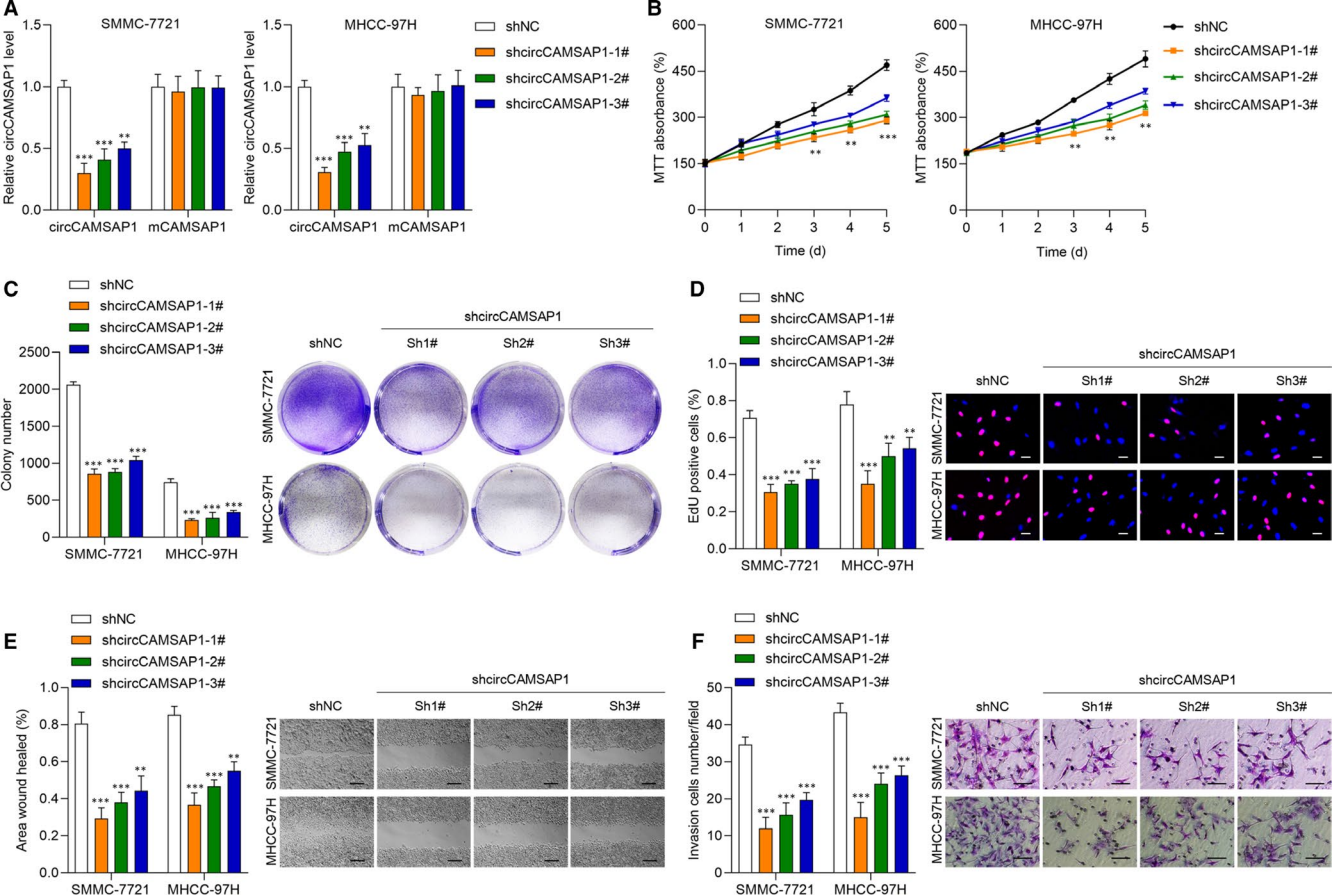
Knockdown of circCAMSAP1 inhibits HCC cell proliferation, migration and invasion. A, The knockdown efficiency was determined by qRT‐PCR in SMMC‐7721 and MHCC‐97H. B, MTT assays revealed that down‐regulation of circCAMSAP1 significantly reduced the growth rate. C, Colony formation assay showed that down‐regulation of circCAMSAP1 reduced the mean colony number. Representative images of SMMC‐7721 and MHCC‐97H. D, EdU assays showed that down‐regulation of circCAMSAP1 reduced the proliferation of HCC cells. Representative images of SMMC‐7721 and MHCC‐97H. Scale bars, 20 µm. E, Wound‐healing assays showed that down‐regulation of circCAMSAP1 reduced cell migration rate. Representative images of SMMC‐7721 and MHCC‐97H migration measured at 48 h. Scale bars, 100 µm. F, Transwell invasion assays showed that down‐regulation of circCAMSAP1 reduced cell invasion number. Representative images of SMMC‐7721 and MHCC‐97H invasion across the transwell measured at 48 h. Scale bars, 50 µm. Data are shown as the mean ± standard deviation (n = 3) and representative of three independent experiments. *P < .05. **P < .01. ***P < .001

To uncover the biological function of *circCAMSAP1* in HCC, we constructed *circCAMSAP1* knockdown cell models. The transfection efficiency of sh‐*circCAMSAP1*#1, sh‐*circCAMSAP1*#2, sh‐*circCAMSAP1*#3 and sh‐NC in SMMC‐7721 and MHCC‐97H cell was tested (Figure 2A). Then, we performed MTT, cell colony and EdU assays to verify the effect of *circCAMSAP1* on HCC cell proliferation. As shown in Figure 2B‐D, it was suggested that the down‐regulation of *circCAMSAP1* significantly inhibited HCC cell proliferation ability. Next, a wound‐healing assay was conducted for cell migration ability detection. *CircCAMSAP1* down‐regulation obviously reduced HCC cell migration ability (Figure 2E). Cell invasion ability was also detected by performing Transwell assay, and it was found that circCAMSAP1 down‐regulation inhibited HCC cell invasion. Collectively, circCAMSAP1 might function as an important role in HCC biological progression.

### 3.3 CircCAMSAP1 functions as a sponge for miR‐1294

**FIGURE 3:**
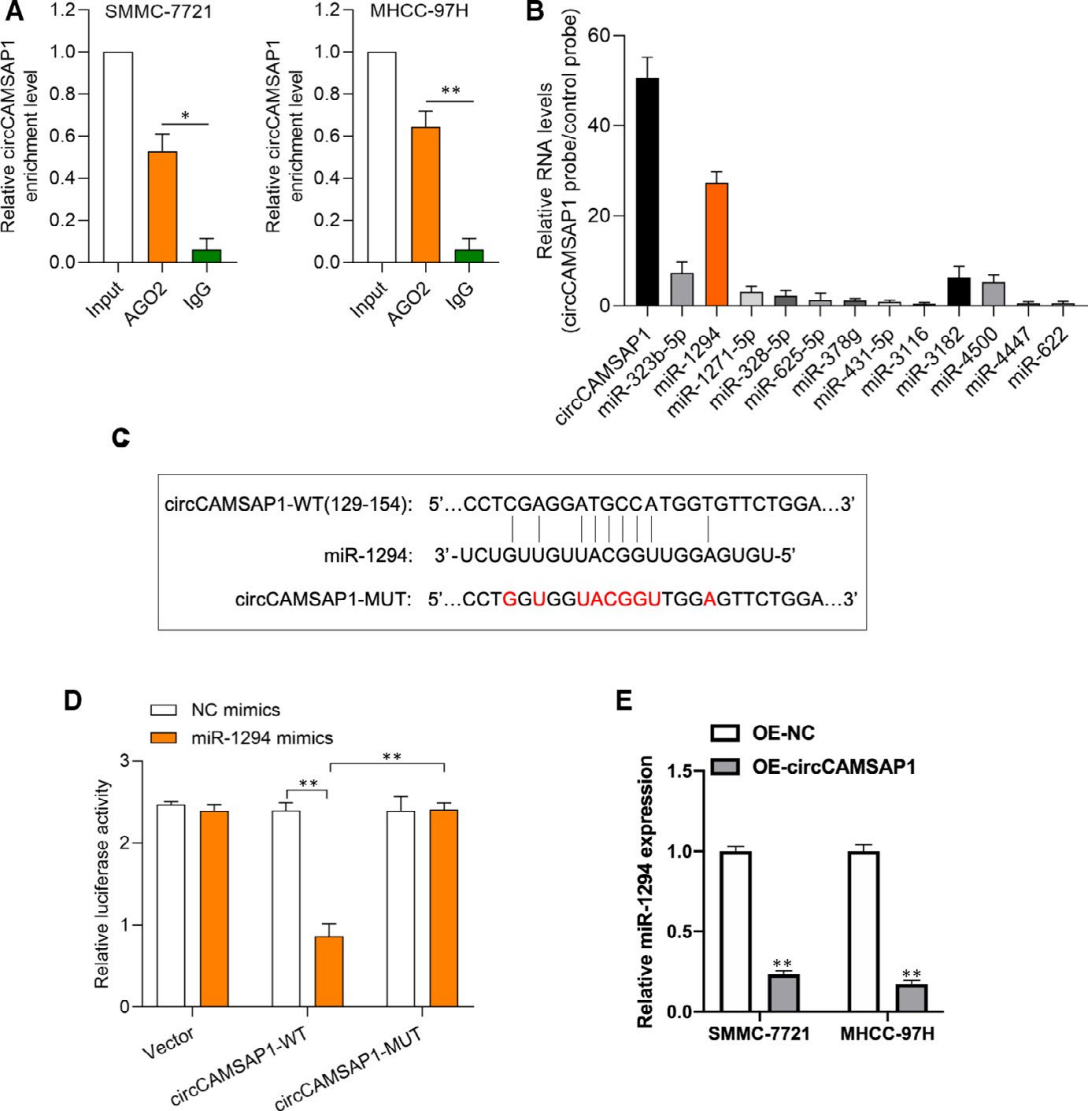
CircCAMSAP1 functions as a sponge for miR‐1294. A, RNA immunoprecipitation (RIP) experiments were performed using an antibody against AGO2 on extracts from HCC cells, to analyse the binding of circCAMSAP1 to the AGO2 protein. B, qRT‐PCR assays were performed to analyse potential miRNAs associated with circCAMSAP1. The enrichment of circCAMSAP1 and microRNAs was detected by RT‐qPCR and normalized to the control probe. C, Wild‐type (WT) and mutated‐type (MUT) sequences of the putative binding sites between circCAMSAP1 and miR‐1294. D, SMMC‐7721 cells were cotransfected with dual‐luciferase reporter vectors and miR‐1294 mimics or negative control mimics (NC mimics). E, Relative expression of miR‐1294 in OE‐NC or OE‐circCAMSAP1 transfected SMCC‐7721 and MHCC‐97H cells was measured by qRT‐PCR. All experiments were repeated at least three times. *P < .05. **P < .01. ***P < .001

Next, we investigated the molecular mechanism of *circCAMSAP1* in HCC progression. Anti‐*AGO2* RIP experiment was conducted to verify the microRNA binding possibility of *circCAMSAP1*; as shown in Figure 3A, the enrichment of *circCAMSAP1* was obviously higher in the *AGO2* group than IgG group. By using a *bio‐circCAMSAP1* probe, we conducted circRNA in vivo precipitation (circRIP) assay to analyse potential miRNAs associated with *circCAMSAP1*. It was found that the expression of *miR‐1294* was highest in the enrichment of *circCAMSAP1*, suggesting that *miR‐1294* may bind to *circCAMSAP1* (Figure 3B). The binding sites between *circCAMSAP1* and *miR‐1294* were predicted by TargetScan Human database (http://www.targetscan.org/vert_72/) (Figure 3C). Dual‐luciferase reporter assays were performed to validate the association of *circCAMSAP1* and *miR‐1294* in SMMC‐7721 cells. As shown in Figure 3D, the luciferase activity in *miR‐1294* mimics and luciferase reporter vector harbouring *circCAMSAP1*‐WT sequence plasmids cotransfected SMMC‐7721 cells was significantly lower than other groups. Furthermore, it was found that *circCAMSAP1* negatively regulated *miR‐1294* expression in HCC cells (Figure 3E). Here, the above results revealed that miR‐1294 might participate in HCC progression via binding to circCAMSAP1.

### 3.4 The promotive effect of circCAMSAP1 on HCC progression is rescued by miR‐1294

**FIGURE 4:**
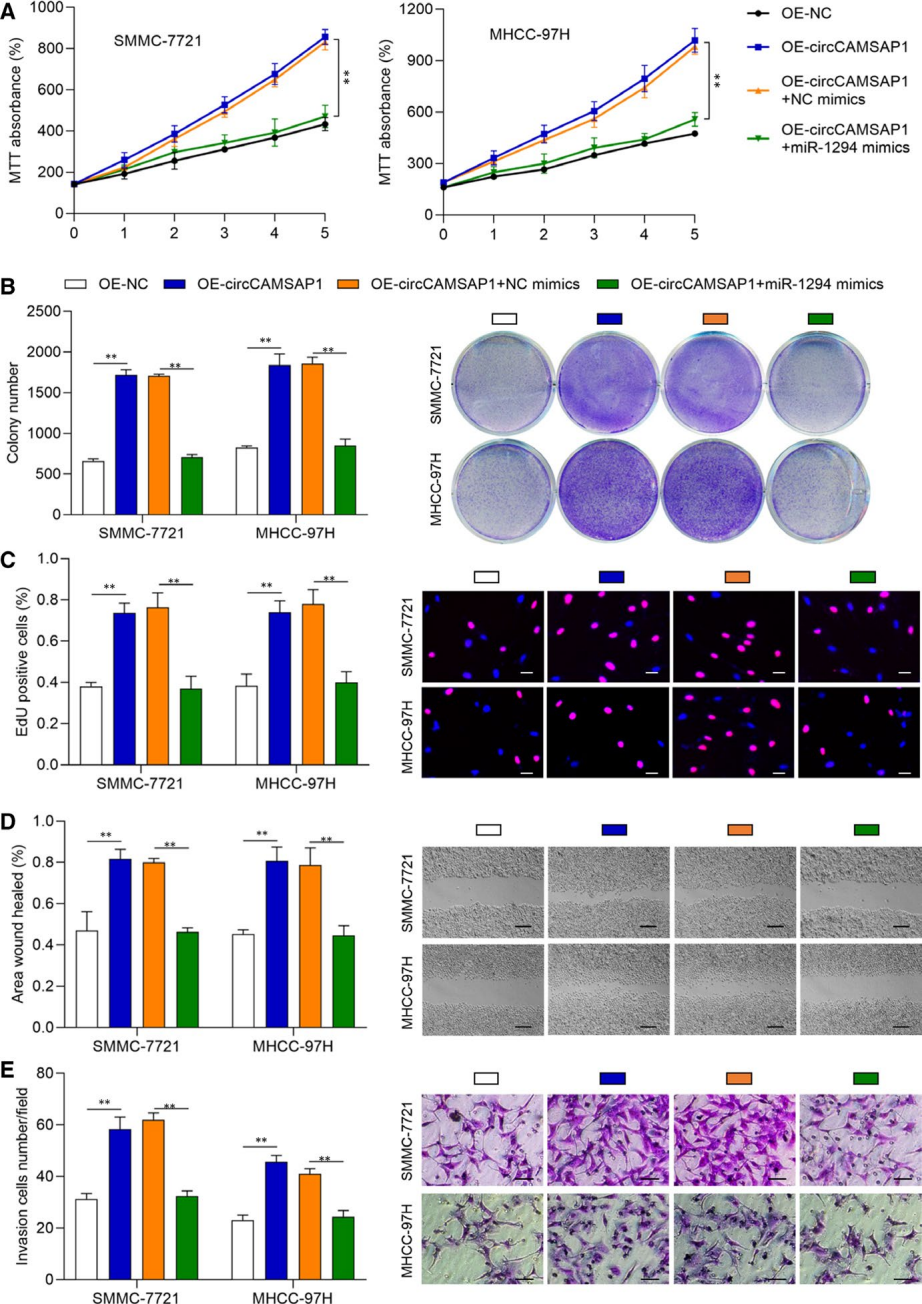
The promotive effect of circCAMSAP1 on HCC progression is rescued by miR‐1294. A, The cell growth rate was increased by overexpression of circCAMSAP1 and rescued by miR‐1294 mimics via MTT assay. B and C, Colony formation assays and EdU assays showed overexpression of circCAMSAP1 promoted HCC cells proliferation and rescued by miR‐1294 mimics. Representative images of SMMC‐7721 and MHCC‐97H. Scale bars, 20 µm. D, Overexpression of circCAMSAP1 promotes cell migration and rescued by miR‐1294 mimics via wound‐healing assay. Representative images of SMMC‐7721 and MHCC‐97H migration measured at 24 h. Scale bars, 100 µm. E, Transwell invasion assay showed that overexpression of circCAMSAP1 increased cell invasion number and rescued by miR‐1294 mimics. Representative images of SMMC‐7721 and MHCC‐97H invasion across the transwell measured at 36 h. Scale bars, 50 µm. Data are shown as the mean ± standard deviation (n = 3) and representative of three independent experiments. *P < .05. **P < .01. ***P < .001

To verify whether *miR‐1294* exerts its function via *circCAMSAP1* in HCC progression, HCC cell models were constructed by transfecting OE‐NC, OE‐*circCAMSAP1*, OE‐*cicrCAMSAP1* + NC mimic and OE‐*cicrCAMSAP1* + *miR‐1294* mimic into SMMC‐7721 and MHCC‐97H cells, respectively. Next, MTT, cell colony and EdU assays were performed to validate the proliferation ability of these cells. As shown in Figure 4A‐C, *circCAMSAP1* promoted cell proliferation ability, but the promotive effects of *circCAMSAP1* were rescued by *miR‐1294* mimic. Moreover, wound‐healing and transwell invasion assays were applied to detect the treated cell migration and invasion ability. It was also found that the promotive effects of *circCAMSAP1* on HCC migration and invasion were rescued by *miR‐1294* mimic (Figure 4D,E). The above results suggested that *circCAMSAP1* promoted HCC progression partially via regulating *miR‐1294*.

### 3.5 CircCAMSAP1 up‐regulates GRAMD1 expression through miR‐1294 to promote HCC progression

**FIGURE 5:**
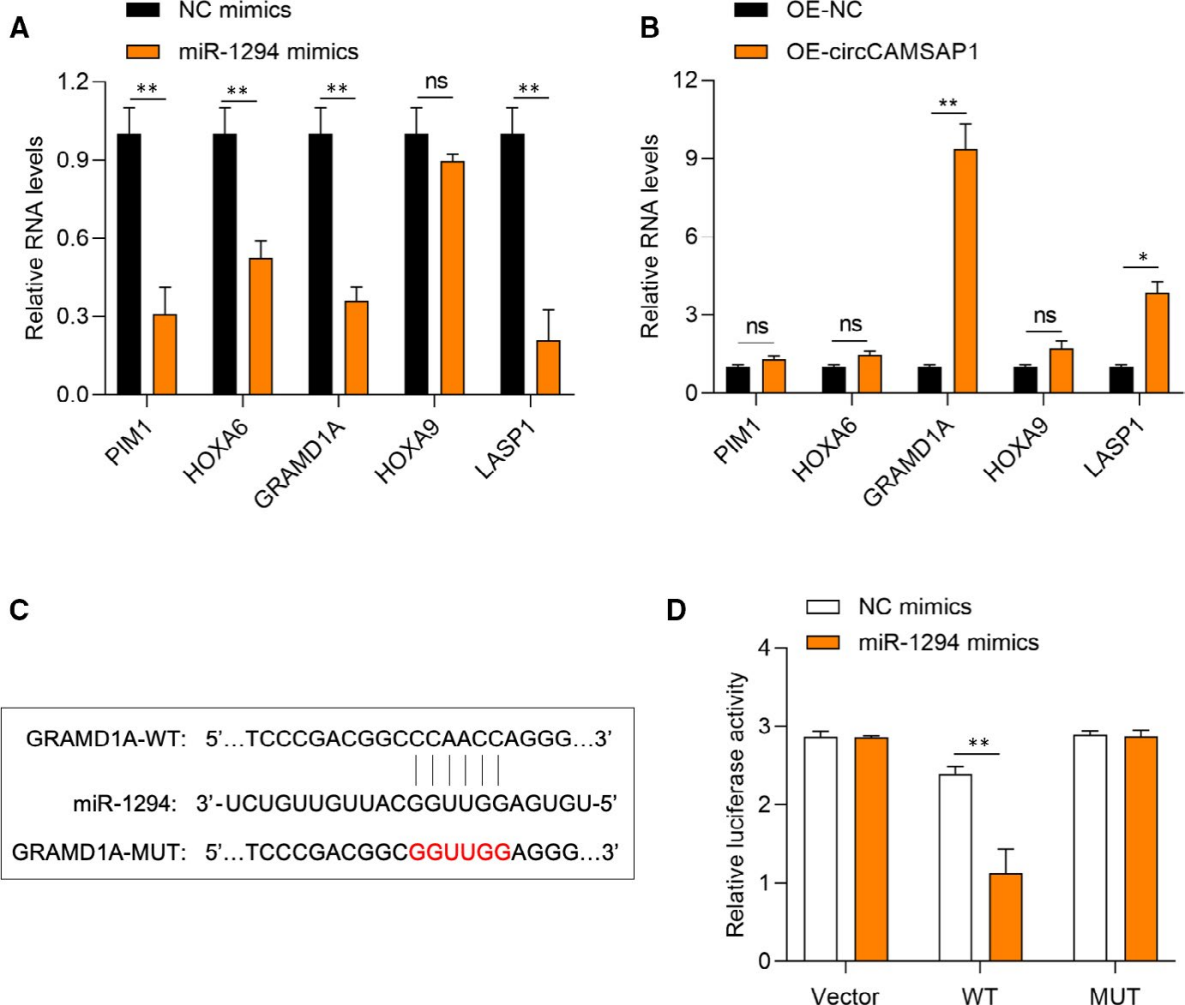
CircCAMSAP1 up‐regulates GRAMD1 expression through miR‐1294 to promote HCC progression. A, The expression of PIM1, HOXA6, GRAMD1A, HOXA9 and LASP1 was down‐regulated upon overexpression of miR‐1294 in SMMC‐7721 cells. GAPDH was used as a control. B, Target gene of miR‐1294 was changed upon overexpression of circCAMSAP1 in SMMC‐7721 cells. C, GRAMD1 wild‐type (WT) and mutated‐type (MUT) sequences of the putative binding sites between GRAMD1 and miR‐1294. D, Dual‐luciferase reporter assays were performed to validate the association of GRAMD1 and miR‐1294. SMMC‐7721 cells were cotransfected with dual‐luciferase reporter vectors and miR‐1294 mimics or negative control mimics (NC mimics). *P < .05. **P < .01

To further explore the function of *miR‐1294* in HCC progression, TargetScan Human database was used to screen its mRNA targets. The predicted targets *PIM1, HOXA6, GRAMD1A, HOXA9* and *LASP1* expression were measured in *miR‐1294*, and *circCAMSAP1* overexpressed SMMC‐7721 cells, respectively. It was found that *GRAMD1A* was down‐regulated upon overexpression of *miR‐1294* but significantly up‐regulated upon overexpression of *circCAMSAP1*, suggesting that *GRAMD1A* might be the target of *miR‐1294* (Figure 5A‐B). The predicted binding sites between *GRAMD1A* and *miR‐1294* by the TargetScan Human database are shown in Figure 5C. Dual‐luciferase reporter assays were performed to validate the association of *GRAMD1* and *miR‐1294*. As shown in Figure 5D, the luciferase activity in *miR‐1294* mimics and luciferase reporter vector harbouring *GRAMD1A*‐WT sequence plasmids cotransfected SMMC‐7721 cells was obviously lower. It was suggested that *circCAMSAP1* promoted HCC progression partially via the *miR‐1294/GRAMD1A* axis.

### 3.6 Down‐regulation of CircCAMSAP1 inhibits the growth of HCC cells in vivo

**FIGURE 6:**
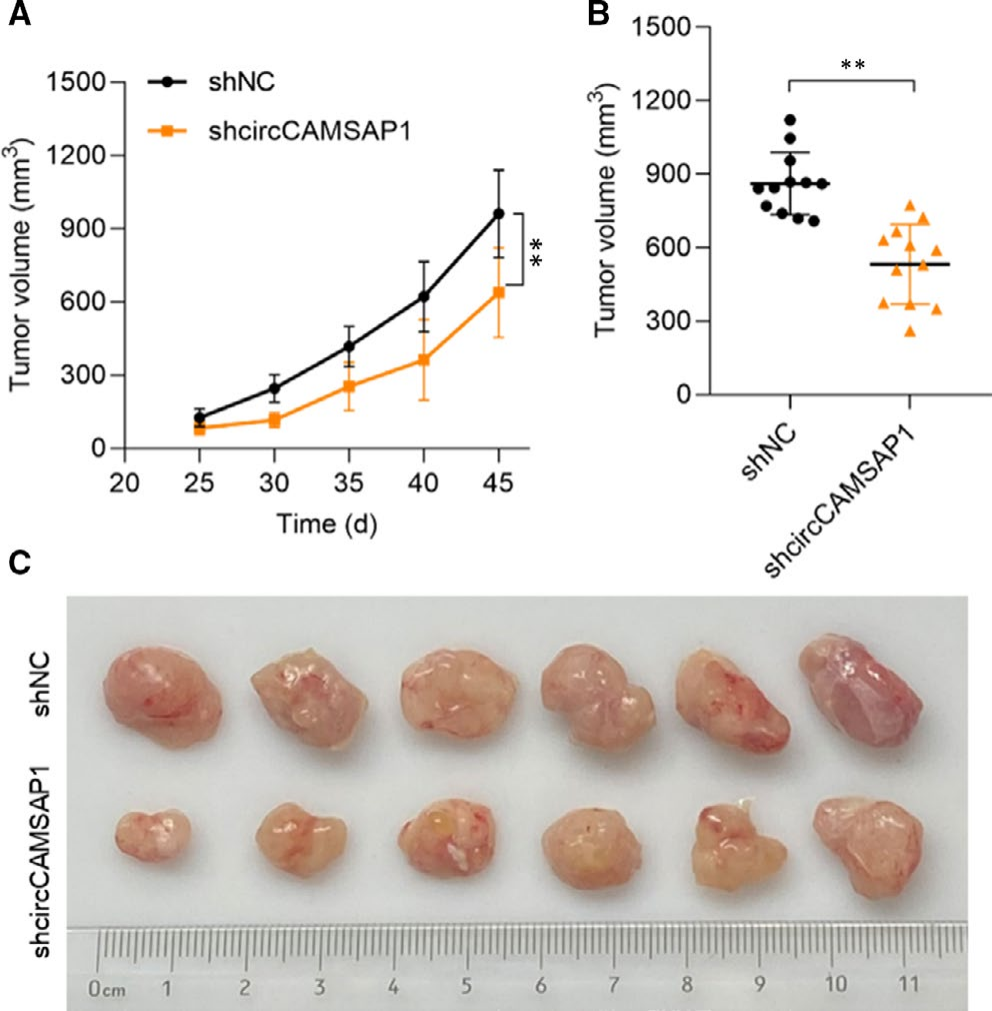
Down‐regulation of CircCAMSAP1 inhibits the growth of HCC cells in vivo. A, NOD/SCID mice (8 wks old) were inoculated subcutaneously with SMMC‐7721 cells (1 × 10^7^ per tumour) and pre‐transfected with sh‐NC or sh‐circCAMSAP1. Tumour volumes were measured as indicated. Data points are presented as mean volume ± SD values. B and C, Comparative statistics of tumour end volumes and images of excised tumours from six NOD/SCID mice at 45 d. All experiments were repeated at least three times. **P < .01 (Student's *t* test)

Here, to further confirm the function of *circCAMSAP1* in HCC progression, in vivo experiment was applied. NOD/SCID mice (8 weeks old) were inoculated subcutaneously with SMMC‐7721 cells (1 × 10^7^ per tumour) and pre‐transfected with sh‐NC or sh‐*circCAMSAP1*. As shown in Figure 6A, xenograft tumour volumes were measured 5 days a time from day 25 to 45, and down‐regulation of *circCAMSAP1* significantly inhibits the growth of HCC cells in vivo. Comparative statistics of tumour end volumes are shown in Figure 6B. The representative images of excised tumours are presented in Figure 6C. Our results suggested that *circCAMSAP1* regulated HCC progression both in vitro and in vivo.

## 4 DISCUSSION

In the present study, our study found that the expression of *circCAMSAP1* was up‐regulated in HCC tissues compared with its adjacent normal tissues. It has been reported that *circSETD, circRNA‐104075 and circRNA‐104718* ^28^, ^29^, ^30^ expression is up‐regulated in HCC tissues and regulates HCC biological functions, such as proliferation, migration, invasion and apoptosis, indicating the important role of circRNAs in HCC development. Zhou et al demonstrated that *circCAMSAP1* is up‐regulated in colorectal cancer tissues and promotes the malignant behaviours of colorectal cancer,^24^ whereas, whether *circCAMSAP1* contributes to the progression of HCC remains uncovered. Here, we assumed that dysregulated *circCAMSAP1* might affect HCC cellular progression. By performing MTT, cell colony, EdU, wound‐healing and transwell invasion assays, our findings suggested that knockdown of circCAMSAP1 inhibited HCC cell proliferation, migration and invasion ability.

The molecular mechanisms of circRNAs in the progression of HCC have been wildly studied. It is well known that circRNAs are capable to regulate its downstream miRNA targets through directly sponging on it.^7^, ^31^ Therefore, bioinformatic analysis, AGO2‐RIP, circRIP and luciferase reporter assays were performed. And, circCAMSAP1 was found to act as a molecular sponge for miR‐1294 in HCC cells. The role of miR‐1294 has been found to participate in the development of several cancers via mediating cellular progress.^32^, ^33^, ^34^, ^35^, ^36^ Here, our results found that miR‐1294 mimic rescued the promotive effect of circCAMSAP1 on HCC cell proliferation, migration and invasion, suggesting that circCAMSAP1 regulates HCC tumorigeneses via facilitating miR‐1294. To date, the function of miR‐1294 has not been studied in HCC, and those results indicated that miR‐1294 may play its role in HCC initiation and progression. While, its biological functions and molecular mechanisms demand further study in HCC.

Subsequently, the molecular functions of miR‐1294 were investigated. TargetScan Human database was used to screen the potential targets of miR‐1294, and we measured the expression of the predicted target in miR‐1294 and circCAMSAP1 overexpressed SMMC‐7721 cells. The results suggested that GRAMD1A might be a target for miR‐1294, and a luciferase reporter assay was conducted to assess the association. These results suggested that circCAMSAP1 promotes HCC progression via the miR‐1294/GRAMD1A axis. And the promotive effect of circCAMSAP1 in vivo has also been detected. Moreover, the biological functions of the circCAMSAP1/miR‐1294/GRAMD1A axis in HCC progression remain further investigate in our future study.

Collectively, in this study, we demonstrated the promotive effect of circCAMSAP1 in HCC progression both in vitro and vivo, and we identified circCAMSAP1 acts as a molecular sponge of miR‐1294 and regulates GRAMD1A expression in HCC cell lines. We might provide a potential prognosis marker for HCC and a novel therapeutic target for HCC clinical intervention.

## CONFLICT OF INTEREST

The authors declare that they have no competing interests.

## AUTHOR CONTRIBUTIONS

**Zongjiang Luo:** Conceptualization (equal); Data curation (supporting); Formal analysis (supporting); Investigation (supporting); Methodology (supporting); Software (supporting); Validation (supporting); Visualization (supporting). **Libai Lu:** Data curation (supporting); Formal analysis (supporting); Investigation (supporting); Methodology (supporting); Software (supporting); Validation (supporting); Visualization (supporting). **Qianli Tang:** Conceptualization (supporting); Data curation (supporting); Formal analysis (supporting); Investigation (supporting); Methodology (supporting); Software (lead); Validation (lead); Visualization (lead). **Wang Wei:** Data curation (supporting); Formal analysis (lead); Investigation (supporting); Methodology (supporting); Software (lead); Validation (lead); Visualization (supporting); Writing‐original draft (supporting). **Pengyu Chen:** Data curation (equal); Formal analysis (equal); Investigation (equal); Methodology (equal); Software (equal); Validation (equal). **Yichen Chen:** Data curation (equal); Formal analysis (equal); Software (equal). **Jian Pu:** Conceptualization (lead); Funding acquisition (lead); Methodology (lead); Project administration (lead); Resources (lead); Supervision (lead); Writing‐original draft (lead); Writing‐review & editing (lead). **Jianchu Wang:** Conceptualization (supporting); Data curation (lead); Formal analysis (lead); Investigation (lead); Methodology (lead); Visualization (supporting); Writing‐original draft (supporting).

## Data Availability

All data are included in the article.
